# Hospital ward transitions and outcomes in critical care survivors: A multi-hospital cohort study

**DOI:** 10.1177/17511437261470611

**Published:** 2026-07-28

**Authors:** Toby Falodun, John Masterson, Nazir I. Lone

**Affiliations:** 1 College of Medicine and Veterinary Medicine, University of Edinburgh, UK; 2 Usher Institute, The University of Edinburgh, UK

**Keywords:** critical care, hospital ward transitions, transitions of care, clinical outcomes, cohort study

## Abstract

**Background:**

Hospital ward transitions remain poorly understood despite their importance to patient flow and efficiency. Transition patterns are shaped by local policy and pathways, but in critically ill patients, transitions may also accumulate in response to clinical complexity. This study aimed to characterise patterns of ward transitions, identify determinants of ward transition frequency, and evaluate associations with adverse patient outcomes.

**Methods:**

This retrospective cohort study comprised patients admitted through the emergency department (ED) across three acute hospitals in a region of Scotland between 2017 and 2022 who experienced a critical care admission prior to hospital discharge. Ward transition patterns were characterised using descriptive statistics and frequency distributions. Regression analyses were conducted to identify factors associated with transition frequency and to examine associations with 7-day ED reattendance, 30-day emergency hospital readmission, 30-day post-discharge mortality, and hospital length of stay.

**Results:**

12,836 critical care survivors were included. In univariable analyses, factors including age, comorbidity burden, smoking status, deprivation, and level of organ support were associated with transition frequency. Greater transition burden was associated with increased odds of 7-day ED reattendance (adjusted OR = 1.12, 99.89% CI: 1.07–1.17) and prolonged length of stay (adjusted beta coefficient 1.35, 99.89% CI: 1.34–1.36), but not 30-day readmission (adjusted OR = 1.01, 99.89% CI: 0.96–1.05) or 30-day mortality (adjusted OR = 0.99, 99.89% CI: 0.86–1.14).

**Conclusion:**

Increased ward transitions in critically ill patients mark clinical complexity. It is associated with longer hospital stays and may help identify those at greater risk of early ED reattendance.

## Introduction

Hospital services constitute a significant proportion of National Health Service expenditure, with a substantial increase in future costs expected to come from hospital-based care.^1,2^ Among hospitalised patients, those requiring intensive care unit (ICU) level care require greater resource at roughly twice the daily cost of a standard bed and treatment.^3^ Critically ill patients are a complex and costly population, not only during the acute phase of critical care admission but also as they may experience lengthy recovery periods, sequelae from complications and long-term disability.^4,5^ As a result of their evolving, multidisciplinary needs, many of these patients move through multiple transitions across different hospital wards as they recover.

Specific ward transition patterns are shaped by local policy and pathways and will differ significantly geographically. However, appropriate ward transitions are broadly essential for matching care intensity to clinical acuity, such as escalation to critical care or step-down to rehabilitation. Frequency and pattern of transitions may serve as a meaningful reflection of the demands and trajectory of a patient’s illness. Patients who accumulate significantly more ward transitions throughout their inpatient stay may represent a systematically more complex and vulnerable critically ill population.

In this context, ward transitions could provide a practical and accessible marker of care demands in critically ill patients. By examining the dynamics and consequences of ward transitions, health systems can be designed to identify and anticipate the needs of the critical care survivors, informing resource planning and care design.

Current research relating to hospital ward transitions has predominantly focused on discrete transition points rather than comprehensive patient journeys. Liaison nurse involvement and standardised handover have been associated with improvements in ICU-to-ward transfers.^6,7^ However, research examining patients’ complete trajectories through hospital systems remains relatively scarce. Significant gaps remain in understanding how multiple transitions combine to interact with patient demographics and outcomes.

The primary aim of this study was to evaluate variation in care pathways and its impact on outcomes among critical care patients who survive to discharge. This study focussed on patients admitted via the Emergency Department (ED) as this represents a high-acuity entry point into inpatient care. The study’s specific objectives were: (1) to investigate ward transitions, by analysing their frequency and sequencing; (2) to identify factors that predict the number of ward transitions; (3) and to evaluate associations between ward transitions and 7- ED reattendance, 30-day emergency hospital readmission, 30-day post-discharge mortality and hospital length of stay.

## Methods

This retrospective cohort study used linked healthcare data. Ethical and governance approvals were granted through the DataLoch service (ref DL-2023-036). Pseudonymised patient data was accessed through the DataLoch secure data environment. Statistical analyses were conducted using STATA version 14.1 (Stata Corp, College Station, TX, USA).

The study integrated NHS Lothian patient data from four key data sources: TRAKCare electronic health records (EHRs) from ED and inpatient settings; WardWatcher critical care records from the Scottish Intensive Care Society Audit Group (SICSAG); Scottish Morbidity Records for inpatients and day cases (SMR01); and National Records Scotland’s (NRS) statutory death records.

Patients were eligible for inclusion if they were aged 18 years or older and were admitted to a critical care unit during their hospital stay between 01/01/2017 and 31/12/2022 following an initial ED visit and survived to hospital discharge. Both high dependency units (HDUs) and intensive care units were classified as critical care. The population was restricted to patients admitted to one of the three acute NHS Lothian hospitals: Royal Infirmary of Edinburgh, St John’s Hospital and the Western General Hospital. Patients who were subsequently transferred to other hospitals within NHS Lothian were retained to preserve the full temporal pathway of ward transitions. Exclusion criteria included missing or incomplete admission and discharge data and incomplete ward transition records. Multiple critical care admissions within the same hospitalisation were considered part of the same patient journey and not treated as separate events.

The primary variable of interest was the number of ward transitions during each patient’s hospital stay. Time-stamped ward data from TRAKCare were combined with SMR01 admission and discharge dates to follow patient movement throughout their hospital journeys. The first inpatient ward after a patient’s presentation to an ED was classified as the first ward transition, with every additional ward after that adding to the count.

Wards were grouped into six categories: admissions units, critical care wards, medical wards, surgical wards, and other miscellaneous wards including obstetrics and psychiatric care. Discharge lounges were excluded from the transition count. These ward groupings were used to create a ward sequence variable in which each ward in a patient’s care pathway was chronologically ordered and the ward categories were truncated together. These sequences were reviewed and clinically validated to reflect plausible care trajectories.

Thirteen sociodemographic characteristics, previous health status, and critical care related variables were included to serve as covariates. This included variables regarding age, sex, hospital of admission, main diagnoses, comorbidity count, body mass index, smoking status, electronic frailty index, Scottish Index of Multiple Deprivation, COVID-19 pandemic phase, highest respiratory support, days on vasopressors and days with renal replacement therapy. The critical-care related variables serve as approximate proxies of illness severity.

Main diagnoses were defined based on groupings of International Classification of Diseases, Tenth Revision (ICD-10) codes, with the least prevalent conditions grouped into an ‘Other’ category. Comorbidities were identified using ICD-10 codes mapped to the Charlson Comorbidity Index as described by Hedetoft et al.^8^ (see Table S1). Based on admission date, patients were grouped into four time periods representing distinct phases of NHS operational disruption during the COVID-19 pandemic. The pre-pandemic period (01/01/2017–22/03/2020) precedes notable disruption and the introduction of restrictions.^9^ The peak disruption period (23/03/2020–08/08/2021) encompassed the first wave and national lockdown through multiple restriction cycles and sustained structural reorganisation of NHS services.^10,11^ The transitional period (09/08/2021–20/03/2022) began following Scotland’s move beyond most legal restrictions and the launch of the NHS Recovery Plan^12,13^; despite subsequently encompassing the Delta surge and Omicron wave, with some restrictions briefly reintroduced, hospital admissions remained below earlier pandemic peaks.^14,15^ The post-restriction period (21/03/2022–31/12/2022) followed the removal of nearly all remaining legal COVID-19 restrictions in Scotland and with NHS services operating to address pandemic-related backlogs.^15,16^

The primary outcomes evaluated were: 7-day ED reattendance, 30-day emergency hospital readmission, and 30-day post-discharge mortality. Hospital length of stay was also evaluated as a secondary outcome. However, in contrast to the primary outcomes, number of transitions does not necessarily temporally precede hospital length of stay and so the direction of association cannot be established. TRAKCare data was used to identify 7-day ED reattendance. Hospital length of stay and 30-day emergency hospital readmission was determined using SMR01 data. 7-day ED reattendance, 30-day emergency hospital readmission, and 30-day post-discharge mortality are defined from the date of discharge.

A baseline characteristics table is reported, comparing the thirteen patient characteristics, later used as covariates, across patients with low (1–3) and high (4+) numbers of ward transitions. A matrix detailing all the individual ward transition patterns and their relative proportions was created; this summarised the frequency of each pairing of patient movement by ward category. Care pathway sequences detailed the exact order of wards in each patient’s care pathway. The twenty most common ward sequences were presented.

Univariable Poisson regression was performed for each covariate to evaluate associations with number of ward transitions. This was followed by multivariable Poisson regression including all thirteen covariates in a single model. Age was scaled in decades (age divided by 10) to improve interpretability. Regression coefficients were exponentiated to report incidence rate ratios (IRRs) with corresponding confidence interval (CI). Patients with missing sociodemographic, health status or critical care related data were handled using the missing indicator method to preserve sample size for regression models.

Binary outcome variables were analysed using logistic regression and reported with odds ratios. Linear regression was used to assess association between ward transitions and length of stay. Length of stay was logarithmically transformed to address its extreme right skew. The log-transformed coefficients were exponentiated to facilitate interpretation, representing multiplicative effects on the original scale. Univariable analyses were performed for each outcome, followed by multivariable models adjusting for the thirteen patient characteristics as covariates.

Effect modification analyses were carried out, evaluating whether the association of ward transitions with outcomes varied based on groupings of age (quartiles), sex and comorbidity count (no comorbidity vs 1 or more comorbidities). Stratified analyses were conducted, and overall interaction terms generated to evaluate differential effects.

To account for the multiple comparisons across the main analysis, Bonferroni correction was applied. Forty-six different tests across all the regression model in the main analyses were performed, adjusting the significance threshold to *p* < 0.0011 (0.05/46) and the corresponding confidence interval to 99.89%.

Two sensitivity analyses were conducted to confirm robustness of the models testing the association of ward transitions with outcomes. First, the analysis was restricted to only patients in the pre-pandemic period and post-restriction period to confirm the results were not heavily influenced by the COVID-19 pandemic. Second, patient characteristics with greater than 5% missing data were excluded as covariates, to confirm that missing data in covariates was not materially affecting the results.

This study was reported in accordance with the Strengthening the Reporting of Observational Studies in Epidemiology (STROBE) guidelines.^17^

## Results

Between 01/01/2017 and 31/12/2022, 355,579 hospital admissions from the ED with complete inpatient records were identified (see Figure S1). Of these, 12,836 were identified with at least one matched WardWatcher record documenting a critical care stay and survived to discharge. These patients were used as the primary population for this study.

The median age for this cohort was 59 with an interquartile range of 40–69. Most patients were admitted to the RIE site, making up over two thirds of the patients. Diagnoses varied across different organ systems, but the most common pathologies were injury and poisoning related (21.6% of total patients). Table 1 details the baseline characteristics of the patients stratified by the number of ward transitions during their inpatient stay.

**Table 1.** Baseline characteristics table.

	Lower (1–3) transitions	Higher (4+) transitions	Total	*p*-Value
Characteristics	*N* = 7501)	*N* = 5335	*N* = 12,836	
Age at attendance to A&E (years)^a^	55 (40, 69)	63 (50, 74)	59 (44, 71)	<0.001
Age at attendance to A&E (years)				<0.001
17–44	2305 (30.7%)	917 (17.2%)	3222 (25.1%)	
45–64	2734 (36.4%)	1978 (37.1%)	4712 (36.7%)	
65–74	1342 (17.9%)	1217 (22.8%)	2559 (19.9%)	
75–84	871 (11.6%)	961 (18.0%)	1832 (14.3%)	
85+	249 (3.3%)	262 (4.9%)	511 (4.0%)	
Sex				0.059
Female	3079 (41.0%)	2279 (42.7%)	5358 (41.7%)	
Male	4422 (59.0%)	3056 (57.3%)	7478 (58.3%)	
Hospital				<0.001
Royal infirmary of Edinburgh	5423 (72.3%)	3383 (63.4%)	8806 (68.6%)	
St John’s Hospital at Howden	840 (11.2%)	811 (15.2%)	1651 (12.9%)	
Western general hospital	1238 (16.5%)	1141 (21.4%)	2379 (18.5%)	
Main diagnosis				<0.001
Infectious	387 (5.2%)	305 (5.7%)	692 (5.4%)	
Neoplasm	268 (3.6%)	388 (7.3%)	656 (5.1%)	
Endocrine	396 (5.3%)	93 (1.7%)	489 (3.8%)	
Neurological	140 (1.9%)	111 (2.1%)	251 (2.0%)	
Circulatory	830 (11.1%)	957 (17.9%)	1787 (13.9%)	
Respiratory	1029 (13.7%)	495 (9.3%)	1524 (11.9%)	
Digestive	1148 (15.3%)	1462 (27.4%)	2610 (20.3%)	
MSK	50 (0.7%)	116 (2.2%)	166 (1.3%)	
Genitourinary	300 (4.0%)	237 (4.4%)	537 (4.2%)	
Other	479 (6.3%)	247 (4.6%)	726 (5.8%)	
Miscellaneous	443 (5.9%)	187 (3.5%)	630 (4.9%)	
Injury, poisoning or other external cause	2031 (27.1%)	737 (13.8%)	2768 (21.6%)	
Comorbidities				<0.001
None	5498 (73.3%)	3678 (68.9%)	9176 (71.5%)	
1 comorbidity	1737 (23.2%)	1359 (25.5%)	3096 (24.1%)	
2 or more comorbidities	266 (3.5%)	298 (5.6%)	564 (4.4%)	
Body Mass Index (kg/m^2^)				<0.001
Underweight (<18.5)	446 (5.9%)	347 (6.5%)	793 (6.2%)	
Normal (18.5–24.9)	2397 (32.0%)	1693 (31.7%)	4090 (31.9%)	
Overweight (25.0–29.9)	1884 (25.1%)	1499 (28.1%)	3383 (26.4%)	
Obese (30.0–39.9)	1491 (19.9%)	1243 (23.3%)	2734 (21.3%)	
Severely obese (>39.9)	404 (5.4%)	271 (5.1%)	675 (5.3%)	
Missing	879 (11.7%)	282 (5.3%)	1161 (9.0%)	
Smoking status				<0.001
Non-smoker	1198 (16.0%)	912 (17.1%)	2110 (16.4%)	
Previous smoker	2066 (27.5%)	1719 (32.2%)	3785 (29.5%)	
Current smoker	1670 (22.3%)	942 (17.7%)	2612 (20.3%)	
Missing	2567 (34.2%)	1762 (33.0%)	4329 (33.7%)	
Electronic Frailty Index (EFI)				<0.001
Fit (efi < 0.12)	2329 (31.0%)	1765 (33.1%)	4094 (31.9%)	
Mildly frail (efi 0.12–0.23)	1556 (20.7%)	1243 (23.3%)	2799 (21.8%)	
Moderately frail (efi 0.24–0.35)	582 (7.8%)	482 (9.0%)	1064 (8.3%)	
Severely frail (efi > 0.35)	181 (2.4%)	149 (2.8%)	330 (2.6%)	
Missing	2853 (38.0%)	1696 (31.8%)	4549 (35.4%)	
SIMD quintiles				<0.001
1 – Most deprived	1449 (19.3%)	850 (15.9%)	2299 (17.9%)	
2	2090 (27.9%)	1448 (27.1%)	3538 (27.6%)	
3	1177 (15.7%)	989 (18.5%)	2166 (16.9%)	
4	1157 (15.4%)	819 (15.4%)	1976 (15.4%)	
5 – Least deprived	1507 (20.1%)	1161 (21.8%)	2668 (20.8%)	
Missing	121 (1.6%)	68 (1.3%)	189 (1.5%)	
COVID–19 period				0.043
Pre-pandemic period	3041 (57.0%)	4325 (57.7%)	7366 (57.4%)	
Peak disruption period	1190 (22.3%)	1598 (21.3%)	2788 (21.7%)	
Transitional period	528 (9.9%)	701 (9.3%)	1229 (9.6%)	
Post-restriction period	576 (10.8%)	877 (11.7%)	1453 (11.3%)	
Highest respiratory support				<0.001
No support	4980 (66.4%)	3235 (60.6%)	8215 (64.0%)	
Non-invasive support	828 (11.0%)	531 (10.0%)	1359 (10.6%)	
Invasive support	1693 (22.6%)	1569 (29.4%)	3262 (25.4%)	
Vasopressor use				<0.001
No	5913 (78.8%)	3331 (62.4%)	9244 (72.0%)	
Yes	1588 (21.2%)	2004 (37.6%)	3592 (28.0%)	
Number of days on vasopressors^a^	0 (0, 0)	0 (0, 2)	0 (0, 1)	<0.001
Renal replacement therapy use				0.033
No	6953 (92.7%)	4997 (93.7%)	11,950 (93.1%)	
Yes	548 (7.3%)	338 (6.3%)	886 (6.9%)	
Number of days on renal replacement therapy^a^	0 (0, 0)	0 (0, 0)	0 (0, 0)	0.067

Includes sociodemographic factors, health status indicators and critical care interventions. Age presented as both continuous and categorical, and other variables as counts and percentages. Stratified by the number of ward transitions (1–3 vs 4 or more).

a Age, number of days on vasopressors and number of days on renal replacement therapy are reported as median (p25, p75).

The number of ward transitions varied across patients in our cohort. Overall, patients experienced a median of 3 transitions (IQR: 2–4; mean 3.51, SD 1.92). As shown in Table 2, nearly 50% of patients were directly admitted to a critical care unit from the ED. Patients who experienced an additional ward transition prior to critical care admission were primarily directed through admissions units and surgical wards.

**Table 2.** Transition matrix detailing the number of ward-to-ward connections.

Ward type	Admissions (%)	Critical care (%)	Medical (%)	Other (%)	Surgical (%)	Discharge (%)
ED	3102 (24.17)	6278 (48.91)	1034 (8.06)	195 (1.52)	2227 (17.35)	N/A
Admissions	24 (0.59)	1260 (31.21)	1261 (31.24)	44 (1.09)	1010 (25.02)	438 (10.80)
Critical care	681 (3.94)	2815 (16.31)	5359 (31.04)	73 (0.42)	7090 (41.07)	1246 (7.22)
Medical	83 (0.86)	2051 (21.32)	1230 (12.79)	391 (4.07)	1055 (10.97)	4808 (49.99)
Surgical	45 (0.35)	4762 (36.52)	627 (4.81)	328 (2.52)	1506 (11.55)	5770 (44.26)
Other	102 (8.96)	98 (8.61)	107 (9.40)	107 (9.40)	150 (13.18)	574 (50.44)

The rows represent the ward category the patient moved from. The columns represent the ward category the patients moved to. The percentages represent the proportion of ward transitions to each ward category when coming from each ward category.

The most common ward sequences involved direct critical care admission followed by a step down to a single medical or surgical ward once the critical care stay had concluded (Table 3). Other common ward sequences tended to be straightforward with a single ward transition before critical care admission and after critical care admission. After the first few common sequences, the frequency of subsequent ward sequences declined sharpy; however, the large number of rarer sequences collectively made up a substantial portion of all patient trajectories. Overall, the 20 most common ward sequences accounted for just over 60% of ward transition sequences.

**Table 3.** Twenty most common ward sequences.

Most common ward transition sequences	Frequency	Percent	Cum.
Critical care – medical	1872	14.58	14.58
Critical care – surgical	1006	7.84	22.42
Surgical – critical care – surgical	975	7.60	30.02
Critical care	842	6.56	36.58
Admissions – surgical – critical care – surgical	480	3.74	40.32
Admissions – critical care – medical	443	3.45	43.77
Critical care – admissions	364	2.84	46.6
Surgical – surgical – critical care – surgical	255	1.99	48.59
Critical care – critical care – medical	250	1.95	50.54
Medical – critical care – medical	239	1.86	52.4
Admissions – medical – critical care – medical	207	1.61	54.01
Critical care – medical – medical	184	1.43	55.45
Admissions – critical care – surgical	175	1.36	56.81
Medical – critical care – surgical	157	1.22	58.03
Critical care – critical care – surgical	136	1.06	59.09
Admissions – medical – surgical – critical care – surgical	123	0.96	60.05
Critical care – surgical – critical care – surgical	114	0.89	60.94
Medical – surgical – critical care – surgical	101	0.79	61.72
Critical care – admissions – medical	99	0.77	62.5
Admissions – surgical – surgical – critical care – surgical	97	0.76	63.25

Frequency, percentage and cumulative percentage of total ward transition sequences are listed.

In univariable analysis, age, hospital of admission, main condition, comorbidity count, smoking status, Scottish index of multiple deprivation, respiratory support, vasopressor support, and renal replacement therapy had statistically significant associations with the number of ward transitions in patients. Without Bonferroni correction, sex and body mass index also showed significant associations. After adjustment, age, hospital of admission, main condition, respiratory support and vasopressor support remained significantly associated with the number of ward transitions in patients. Without Bonferroni correction, comorbidity count and smoking status also showed significant associations (Table 4).

**Table 4.** Association of ward transitions and patient characteristics.

	Unadjusted		Adjusted	
Characteristic	IRR (99.89% CI)	*p*-Value	IRR (99.89% CI)	*p*-Value
Age/10	1.06 (1.05–1.07)	<0.0001	1.03 (1.02–1.05)	<0.0001
Sex				
Female	1.00		1.00	
Male	0.98 (0.95–1.01)	0.0136	0.99 (0.95–1.02)	0.1272
Hospital of admission				
Royal infirmary of Edinburgh	1.00		1.00	
St John’s Hospital	1.17 (1.12–1.23)	<0.0001	1.18 (1.12–1.24)	<0.0001
Western General Hospital	1.13 (1.09–1.17)	<0.0001	1.05 (1.01–1.10)	<0.0001
Main condition				
Infectious	1.00		1.00	
Neoplasm	1.13 (1.03–1.23)	<0.0001	1.12 (1.11–1.23)	<0.0001
Endocrine	0.71 (0.64–0.80)	<0.0001	0.78 (0.70–0.88)	<0.0001
Neurological	1.04 (0.96–1.18)	<0.3147	1.05 (0.93–1.20)	0.1681
Circulatory	1.14 (1.06–1.23)	<0.0001	1.11 (1.03–1.20)	<0.0001
Respiratory	0.85 (0.78–0.92)	<0.0001	0.88 (0.81–0.96)	<0.0001
Digestive	1.07 (0.99–1.15)	0.0041	1.09 (1.01–1.17)	<0.0001
MSK	1.32 (1.16–1.51)	<0.0001	1.36 (1.19–1.55)	<0.001
Genitourinary	0.96 (0.87–1.06)	<0.1340	0.98 (0.89–1.09)	0.6010
Other	0.89 (0.81–0.97)	<0.0001	0.89 (0.81–0.98)	<0.0001
Miscellaneous	0.84 (0.76–0.92)	<0.0001	0.88 (0.80–0.97)	<0.0001
Injury, poisoning or other external cause	0.81 (0.75–0.87)	<0.0001	0.88 (0.81–0.95)	<0.0001
Comorbidity count				
No comorbidity	1.00		1.00	
1 comorbidity	1.04 (1.00–1.08)	0.0002	1.01 (0.97–1.04)	0.6299
2 or more comorbidities	1.12 (1.04–1.20)	<0.0001	1.05 (0.97–1.13)	0.0328
Body mass Index (kg/m^2^)				
Underweight (<18.5)	1.01 (0.95–1.08)	0.5667	1.04 (0.97–1.11)	0.0835
Normal (18.5–24.8)	1.00		1.00	
Overweight (25.0–29.9)	1.03 (0.99–1.07)	0.0141	1.00 (0.96–1.04)	0.7993
Obese (30.0–39.9)	1.04 (1.00–1.09)	0.0017	1.01 (0.97–1.06)	0.3640
Severely obese (>39.9)	0.99 (0.92–1.02)	0.6470	0.98 (0.91–1.06)	0.4691
Missing	0.80 (0.75–0.85)	<0.0001	0.87 (0.81–0.92)	<0.0001
Smoking status				
Non-smoker	1.00		1.00	
Previous smoker	1.02 (0.97–1.07)	0.1403	1.00 (0.96–1.05)	0.8450
Current smoker	0.92 (0.87–0.97)	<0.0001	0.97 (0.91–1.02)	0.0294
Missing	0.97 (0.93–1.02)	0.0540	1.02 (0.97–1.07)	0.1413
Electronic Frailty Index				
Fit (efi < 0.12)	1.00		1.00	
Mildly frail (efi 0.12–0.23)	1.01 (0.97–1.06)	0.3396	0.99 (0.95–1.03)	0.3953
Moderately frail (efi 0.24–0.35)	1.03 (0.97–1.09)	0.1178	1.00 (0.94–1.07)	0.9543
Severely frail (efi > 0.35)	1.00 (0.91–1.11)	0.9293	0.99 (0.89–1.09)	0.6717
Missing	0.92 (0.89–0.96)	<0.0001	0.97 (0.94–1.02)	0.0414
Scottish Index of multiple deprivation				
1 – Most deprived	1.00		1.00	
2	1.05 (1.00–1.10)	0.0022	1.01 (0.97–1.06)	0.3136
3	1.08 (1.02–1.13)	<0.0001	1.03 (0.97–1.08)	0.1086
4	1.05 (1.00–1.11)	0.0015	1.01 (0.95–1.06)	0.6362
5 – Least deprived	1.08 (1.03–1.13)	<0.0001	1.00 (0.95–1.05)	0.9801
Missing	1.05 (0.92–1.20)	0.2049	1.10 (1.02–1.19)	0.0364
COVID-19 timing				
Pre pandemic	1.00		1.00	
Peak disruption period	1.02 (0.98–1.06)	0.1723	1.01 (0.97–1.05)	0.5187
Transitional period	1.01 (0.96–1.07)	0.3662	0.99 (0.94–1.04)	0.4983
Post-first year of pandemic	0.97 (0.92–1.02)	0.0518	0.95 (0.90–1.00)	0.0014
Highest respiratory support				
No support	1.00		1.00	
Non-invasive ventilation	0.99 (0.94–1.04)	0.6053	1.04 (0.98–1.10)	0.0303
Invasive ventilation	1.14 (1.11–1.19)	<0.0001	1.08 (1.03–1.12)	<0.0001
Days on vasopressors	1.04 (1.04–1.05)	<0.0001	1.04 (1.03–1.04)	<0.0001
Days with renal replacement therapy	1.02 (1.01–1.02)	<0.0001	1.00 (0.99–1.01)	0.2542

Incidence rate ratio (IRR), 95% confidence interval and *p*-value for unadjusted and adjusted models are shown.

11.12% of patients in the cohort reattended to the ED within 7 days of discharge. Among patients who experienced only 1–3 ward transitions 10.3% experienced reattendance compared to 12.33% of patients with 4 or more ward transitions. 14.9% of patients in the cohort were readmitted through the emergency department at 30-days post discharge. Among patients who experienced only 1–3 ward transitions 14.7% experienced readmission compared to 15.2% of patients with 4 or more ward transitions. 1.53% of patients in the cohort died within 30 days of discharge. Among patients who experienced only 1–3 ward transitions, 1.52% died within 30 days of discharge compared to 1.54% of patients with 4 or more ward transitions.

**Figure 1.**
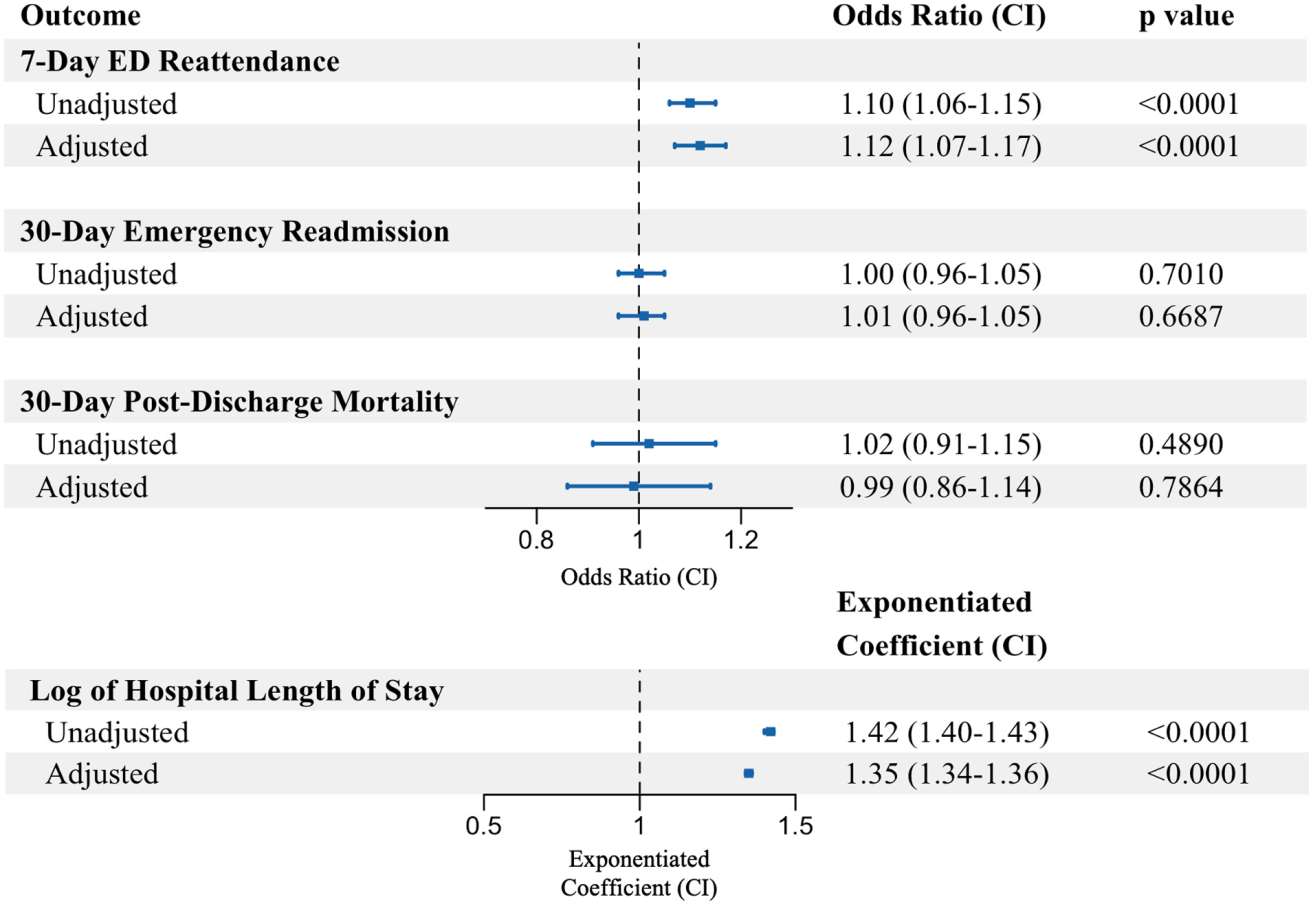
Forest plot of the associations of the number of ward transitions with 7-day emergency department reattendance, 30-day emergency hospital readmission, 30-day post discharge mortality, and log of hospital length of stay. Includes odds ratio (for binary outcomes) and coefficient (continuous outcome) with 99.89% confidence interval and *p*-value. ED: emergency department.

The number of ward transitions was associated with an increased odds of 7-day ED reattendance (unadjusted OR = 1.10, 99.89% CI: 1.06–1.15; adjusted OR = 1.12, 99.89% CI: 1.07–1.17). The number of ward transitions was also found to be significantly associated with prolonged length of stay even after adjustment (unadjusted exp(B) = 1.42, 95% CI: 1.40–1.43; adjusted exp(B) = 1.35, 99.89% CI: 1.34–1.36). However, there was no association with 30-day emergency hospital readmission (unadjusted OR = 1.00, 99.89% CI: 0.96–1.05; adjusted OR = 1.01, 95% CI: 0.96–1.05) or 30-day post discharge mortality (unadjusted OR = 1.02, 99.89% CI: 0.91–1.15; adjusted OR = 0.99, 99.89% CI: 0.86–1.14). See Figure 1 for a forest plot of associations between ward transitions and outcomes.

### Effect modification analyses

We performed subgroup analyses stratified by age quartile, sex and comorbidity burden. For most subgroups, the associations observed in the main analysis were consistent across strata and interaction tests were not statistically significant. However, the association between the number of ward transitions and log length of stay varied by age quartiles (interaction *p* < 0.0001), with the youngest quartile showing a larger magnitude association between the number of ward transitions and log length of stay (adjusted exp(B) = 1.39, 99.89% CI: 1.37–1.42) compared to older adults.

### Sensitivity analyses

Sensitivity analyses produced results consistent with the primary analysis. In the first, patients admitted during the peak disruption and transitional COVID-19 periods were excluded. In the second, BMI, smoking, and frailty were removed as covariates due to high missingness.

## Discussion

Survivors of hospitalisation involving critical care tend to follow through a few common ward sequences. However, a notable minority of patients experienced more complex pathways with additional moves between several surgical and medical wards. These more complex pathways likely reflect a combination of clinical factors, such as changing diagnoses or new complications.

Several factors were identified as predictors of the number of ward transitions. Age, hospital of admission, main condition, respiratory support and vasopressor support remained significantly associated with the number of ward transitions in patients in multivariable models. These factors tend to reflect that sicker and clinically vulnerable patients are more likely to experience a higher number of ward transitions during inpatient stays. These factors tend to be independently associated with poor patient outcomes and high resource use, suggesting that higher numbers of ward transitions are more likely in sicker patients and may be a marker of vulnerable patient groups.

Increased numbers of ward transitions were found to be associated with increased odds of 7-day ED reattendance and prolonged length of hospital stay. These associations are consistent with ward transitions serving as a marker of clinical complexity; more demanding care needs may lead to patients accumulating more transitions and having poorer short-term outcomes after discharge. The association between ward transitions and hospital length of stay were stronger in younger patients. However, the number of ward transitions was not associated with an increased odds of 30-day emergency hospital readmission or 30-day post-discharge mortality. This suggests that increasing ward transitions is not associated with more severe clinical outcomes occurring beyond the acute discharge period. This may also be consistent with a survivor cohort in which the most severely ill patients are less likely to have survived to discharge, effectively selecting for patients whose complexity, though reflected in transition burden, falls below the threshold that drives readmission or mortality.

A high proportion of older adults are known to interact with healthcare systems each year, but most interactions are straightforward and brief.^18^ In this study, ward transitions follow a similar pattern; most patients experience a modest number of transitions with a smaller subgroup experiencing rarer, complex care pathways. Care pathways tended to adhere to a few key ward sequences, likely specific to each condition. Current literature on this topic has explored the outcomes of patients who diverge from the norm for their specific disease. For example, medical patients who spend time in non-medical wards have been shown to experience longer lengths of stay, possibly due to fragmented communication and unfamiliarity with the patient’s condition.^19^ In these atypical care pathways, increased ward transitions are also known to be of poorer quality for similar reasons.^20^

There are numerous known and suspected drivers of care pathways and ward transitions. While existing evidence has primarily focused on adjusting for well-established characteristics like age, ethnicity and comorbidity, it often overlooks other important factors, including aspects related to a patient’s social history.^21–24^ Much of this research was done in broader populations such that factors related to critical care were not a consideration, in contrast to our study.

The existing evidence linking frequent ward transitions to adverse patient outcomes is largely observational and retrospective,^25,26^ and subject to many of the same methodological limitations relevant to the present study. Associations have been reported with increased hospital length of stay, falls, wound infections and greater resource utilisation.^21–24^ However, the directionality of these relationships remains difficult to establish. It is possible that ward transitions are driving poor outcomes as independent risk factors but more plausibly, may be a marker of clinical complexity, a distinction that observational designs are inherently limited in resolving.

Our study has a number of strengths. We were able to leverage granular, time-stamped data from EHRs to evaluate the type and frequency of ward sequences for the entire inpatient hospitalisation for critical patients. The analyses of 7‑day ED reattendance, 30‑day emergency hospital readmission, and 30-day mortality as post‑discharge outcomes offer a temporal advantage because they occur after the acute hospital stay and are not at risk of reverse causality. In addition, these measures more directly reflect the quality of discharge planning, and handovers. In‑hospital metrics such as length of stay, capture the immediate association of ward transitions, but are much less reliable as they are being used for an exposure that is occurring at the same time. Sensitivity analyses were carried out to confirm the validity of results even after accounting for potential bias related to data missingness and the COVID-19 pandemic.

Our study was limited due to the retrospective cohort design. The data were relatively dated and there were moderate amounts of missing sociodemographic and health status data (see Table 1). Our study consists of a population of survivors, so the findings only generalise to ICU survivors rather than all critically ill patients. While readmissions to any of the three acute hospitals within NHS Lothian were captured, information for patients who were subsequently readmitted to hospitals outside of NHS Lothian could not be included in the analysis. Despite adjustment for a range of potential confounders, disease severity was only captured by number and type of organ support during the critical care stay. As illness severity could be a major driver of ward transition patterns and patient outcomes, residual confounding may be present as the dataset lacked sufficient information to calculate granular severity scores (e.g. APACHE II, SOFA) for all patients. Furthermore, we were unable to measure systemic factors which could potentially be mediating the associations between ward transitions and adverse outcomes, including variation across the three hospitals and critical care units in organisational culture, staffing practices, resource availability, post-critical care rehabilitation provision, and follow-up services. These factors may also have varied temporally, especially given the inclusion of the COVID-19 pandemic period within the study timeframe. For these reasons, causal inferences should not be made from our results.

Our study raises considerations for future research and clinical awareness. Future research should ascertain further information relating to transition quality in addition to quantity. This could involve transition-specific measures such as medicine reconciliations and information loss at handovers. Given the descriptive nature of our study, the observed associations between ward transition patterns and 7-day ED reattendance highlight the potential importance of continuity of care during patient transitions. Increased awareness of observed outcome patterns may prompt more meticulous planning in vulnerable patients and future prospective work could examine whether targeted planning at key transition points influences outcomes. Case reviews and morbidity and mortality meetings could incorporate ward transitions information to reflect on how care pathways may have been one of many factors that influenced patient outcomes. Policymakers should consider that most patients experience relatively straightforward care pathways. A notable subset of vulnerable and complex patients experiences far more intricate care pathways. Effective policy might consider atypical care pathways as a target for further investigation and cautious evaluation.

## Conclusion

Frequent ward transitions in hospitalised patients appear to be both a marker of clinical complexity and may have a role in risk stratification for short-term adverse outcomes, including early ED reattendance and prolonged hospitalisation, but are not associated with increased hospital readmission. Future healthcare improvements should focus on deepening understanding of transition processes and their effects on patient outcomes.

## Supplemental Material

sj-docx-1-inc-10.1177_17511437261470611 – Supplemental material for Hospital ward transitions and outcomes in critical care survivors: A multi-hospital cohort study Supplemental material, sj-docx-1-inc-10.1177_17511437261470611 for Hospital ward transitions and outcomes in critical care survivors: A multi-hospital cohort study by Toby Falodun, John Masterson and Nazir I. Lone in Journal of the Intensive Care Society
